# Study of Track Irregularity Time Series Calibration and Variation Pattern at Unit Section

**DOI:** 10.1155/2014/727948

**Published:** 2014-11-04

**Authors:** Chaolong Jia, Lili Wei, Hanning Wang, Jiulin Yang

**Affiliations:** ^1^School of Software Engineering, Chongqing University of Posts and Telecommunications, Chongqing 400065, China; ^2^Chongqing Public Security Bureau, Chongqing 401147, China; ^3^State Key Laboratory of Rail Traffic Control and Safety, Beijing Jiaotong University, Beijing 100044, China; ^4^China National Tendering Center of Mach. & Elec. Equipment, Beijing 100142, China

## Abstract

Focusing on problems existing in track irregularity time series data quality, this paper first presents abnormal data identification, data offset correction algorithm, local outlier data identification, and noise cancellation algorithms. And then proposes track irregularity time series decomposition and reconstruction through the wavelet decomposition and reconstruction approach. Finally, the patterns and features of track irregularity standard deviation data sequence in unit sections are studied, and the changing trend of track irregularity time series is discovered and described.

## 1. Introduction

Time series [1–3] is a statistical method of econometrics. Time series studies the changes showed by observation values of a certain variable in the system in chronological order during a given period and tries to find out the characteristics, future trends and laws over time and the laws are often the consolidated results of impacts by a variety of other factors. Time series does not study the interdependence causality between things, and the study is based on the assumption that some of the information which comes from the historical data can be used to explain the current situation and to predict the future of time series. This reflects an inertia of the development of things with time.

Generally, there are four changing features such as trend [4], periodicity [5], randomness [6], and synthesis [7] in time series and three types of time series prediction including point prediction which offers prediction values, range prediction which offers prediction value within one interval, and density prediction which offers the complete probability distribution of the prediction value.

Time series forecasting is an active study area, and there are a lot of literature on it. In the researching methods of time series forecasting, collection and analysis of historical observations are used to determine the model and to capture the generating process of underlying data, and then the model is used to make prediction. This predictive method is very important in many fields and is widely used in the business, economic [8, 9], industrial [10, 11], engineering [12–14], science [15–21], and other communities. Scholars around the world have been committed to the development and improvement of time series forecasting model in the past few decades.

In the study of time series data, there are some prerequisites for time series modeling in order to make sure the results are accurate and the model is effective. These prerequisites include studying the characteristics of the object data, selecting representative data for study, controlling data quality by means of data correction, analyzing data composition in-depth internally, and discovering implied rules and characteristics in data. All these need to be further studied.

Railway network undertake the important task of passenger and freight traffic; its performance will play an important role for rail transport [22–25]. The railway track states directly determine the safety of train operation. The regularity of the track is not only an important indicator of the track state but also the basis for evaluation of train running quality. Where there is track irregularity, speed limit should be paid attention to; otherwise, in some extreme occasions, overturning might occur. As a result, it is urgent for railway departments to study the law of track irregularity changes so as to master trends of track state changes and to take prevention measures [26, 27]. Various tracks state that inspection data is the most important resource and the accuracy of the data can not only truly reflect the state of the track but also is the basis for modeling and forecasting. Based on the importance of data, this paper identifies abnormal data and calibrates offset data and segment data in order to study track irregularity change trends. In this context, this paper analyses track irregularity data, explores the underlined rules of track irregularity change, predicts future trends, and, ultimately, provides data and models support of track state changes to relevant railway departments, to ensure railway transportation safety.

In this study, track irregularity data is provided by State Key Laboratory of Rail Traffic Control and Safety, Beijing Jiaotong University.

## 2. Abnormal Data and Offset Data

The track irregularity inspection data cannot be found through long-term, continuous monitoring at a certain fixed monitoring site but through the inspection data of various subsections. A single detected data is not time series data, but repeated inspection data is. Meanwhile, each inspection point corresponding to the inspection data will have some offset, which is mainly caused by the inspection device. Since the inspection is dynamic, mileage offset exists in inspection data, so it requires manual correction for every 10 km during the operation of track inspection car. However, there are errors in manual correction, and, according to on-site work experience, this error range is essentially within 50 m, which is still a great error.

Track geometric irregularity data on the timeline at each measuring point should be a time-series data, but in real inspection process, the actual mileage and the mileage measured by track inspection car does not remain the same, and in some occasions the previous measuring points do not correspond to each other, so the result will be as follows: time series data should be constituted by the track irregularity data at the same location but at different time; however, in reality it is constituted by track irregularity data at different time and at different location.

Specifically, mileage offset can be divided into two cases. In the first case, in a single inspection, inspection data and mileage measuring point position correspond to each other accurately, but there are differences between the corresponding measuring points of each time inspection data. In the second case, position of the measuring point corresponding to the inspection data does not correspond with the actual distance, and the actual data is the data corresponding to a position before or after the measuring point. In practice, it is difficult to distinguish these two cases and they can coexist.

## 3. Identify Abnormal Data

Data deviated from the normal value is commonly referred as abnormal data or outliers. In track state inspection process, abnormal inspection data values easily occur due to inspection equipment, locomotives working conditions, and other factors. The anomalies of track irregularity data include two types: overall anomalies and local anomalies.

### 3.1. Overall Abnormal

The track inspection data between October 22, 2007 to June 11, 2008, Beijing-Kowloon line, K500+000–K500+100 unit section is selected as the study object. Outlier curve and normal curve are separated through cluster analysis, and two cluster centers clustering results can be obtained, and outliers track state is detected.

**Figure 1:**
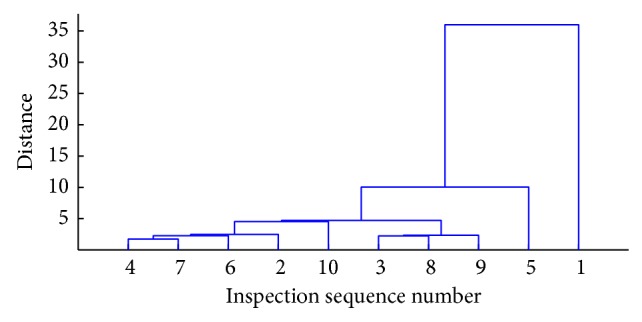
Pedigree chart of clustering.

Pedigree chart of previous gauge irregularity inspection waveform data by cluster analysis is shown in Figure 1.

**Figure 2:**
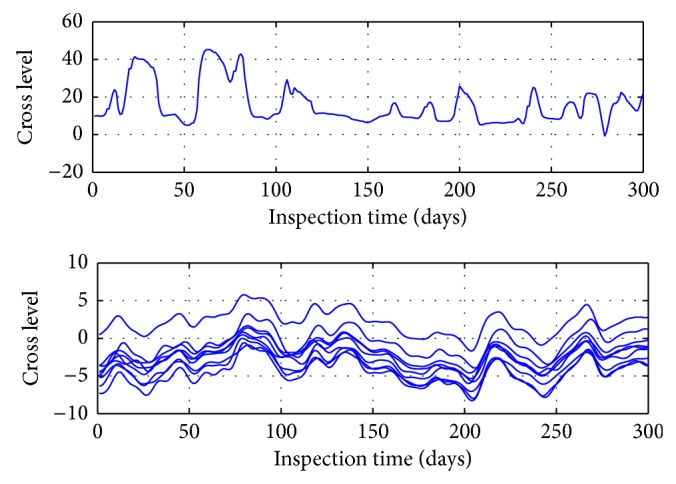
Results of gauge irregularity cluster.

Gauge irregularity cluster results are shown in Figure 2. The following chart is normal data, and the previous chart shows the abnormal value.

### 3.2. Local Anomaly

There will be local phenomenon outliers in track inspection data. In this case, the abnormal data often accounts for a small portion of all the data, but there is a larger difference in amplitude than other normal data.

In recognition of abnormal data, this paper proposes the ratio of difference between track irregularity values at adjacent measuring points to difference between interval lengths at adjacent measuring points (usually roughly 0.25 m). It is defined as an abnormal degree in this paper, and abnormal degree is used to determine and identify outliers' values. The abnormal degree formula is shown as follows:

(1) $$\begin{array}{c} d_{i}=\frac{s_{i}-s_{i-1}}{m_{i}-m_{i-1}}. \end{array}$$

In the formula, *d*_*i*_ is abnormal degree, *s*_*i*_ is track irregularity value at measured point *i*, *s*_*i*−1_ is track irregularity value at measured point *i* − 1, *m*_*i*_ is mileage values of measuring point *i*, and *m*_*i*−1_ is mileage values of measuring point *i* − 1.

**Figure 3:**
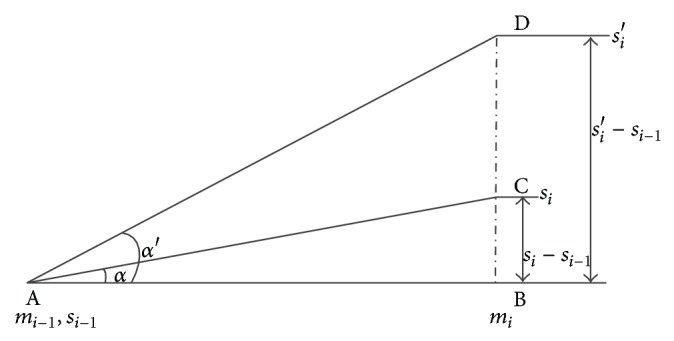
Schematic diagram of track irregularity abnormal state change.

The geometric form of formula (1) is shown in Figure 3. In the formula, abnormality degree is the tangent (*tgα*) in Figure 3. The judgment of track irregularity outlier's recognition is shown in the following.

(*1) Normal Value*. When *tgα* < *k*, it indicates that the state of track irregularity amplitude variations is among the normal range of variation, and in this case, some injuries such as broken rail will not appear.

(*2) Outlier Value*. When *tgα* ≥ *k*, it indicates that the track irregularity state change has exceeded the normal variation amplitude range, and in this case, the track may have serious injuries, such as broken rail.

In Figure 3, *tgα*′ = *k* is the turning point of state exception changes.

**Figure 4:**
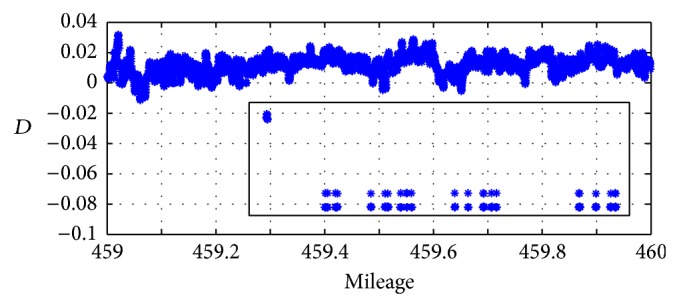
Local outlier values of inspection data in February 23, 2009.

The inspection data of Beijing-Kowloon line in 459 km-460 km mileage section in February 2009 is selected for the study, and the presence of local outliers can be found. The abnormal value of inspection data is shown in Figure 4.

By studying a large number of data, it can be found that, under normal circumstances, most distribution of *d*_*i*_ is [−0.02,0.02]; that is, the range can be set to [−0.02,0.02].

The reasons of the occurrence of abnormal data can be grouped into two categories after analysis: inspection equipment problems (when track inspection car is in abnormal situation, abnormal data will occur); the difference of inspection objects, such as data, when track inspection car through the main line is different from that through turnout.

Abnormal data causes mutations and it must be eliminated. Restoration and correction to abnormal data can improve the effectiveness of the data in analysis, except for the interference of outliers, and then accurate characteristics of track state changing trends can be discovered.

## 4. Abnormal Data Treatment

In case of outliers, there are two measures for treatment: amendment and abandoned.

Deprecated case refers to the situation when data is covered by outliers in large area and the actual value is difficult to be restored and thus has to be abandoned in study. Usually, abandonment is seldom to be seen, and only exception data occurs sometimes. As long as the abnormal data is corrected, it can still be used for research.

**Figure 5:**
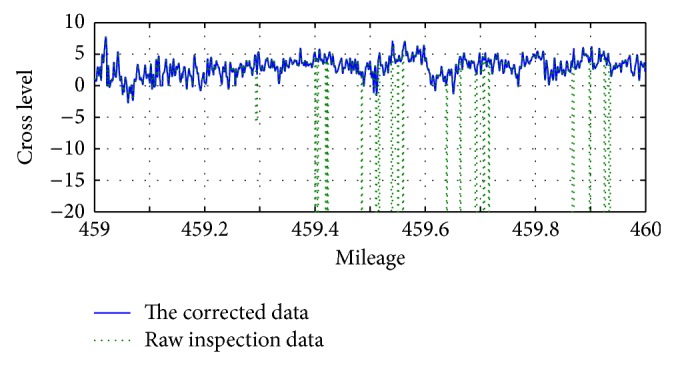
Comparison between revised local outliers data and original value in February 23, 2009.

As the track is continuous physically and spatially, track geometry irregularity changes along mileage direction show continuous features. According to this continuity character, it can be corrected by linear interpolation abnormal data. After correction of outliers, the comparison between the original data and revised local anomaly value in inspection data in February 23, 2009 is shown in Figure 5.

**Figure 6:**
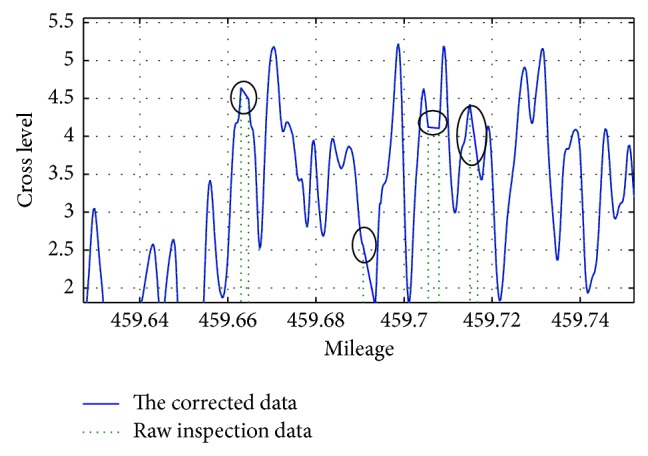
Details of the correction data.

Local details of correction data are shown in Figure 6.

## 5. Data Correction

The practice of using mileage offset data to analyze track state at specified measuring point not only brings large deviation and does not reflect the true state but also is of no significance. So offset correction is needed.

There are two types of data correction: absolute correction and relative correction.

**Figure 7:**
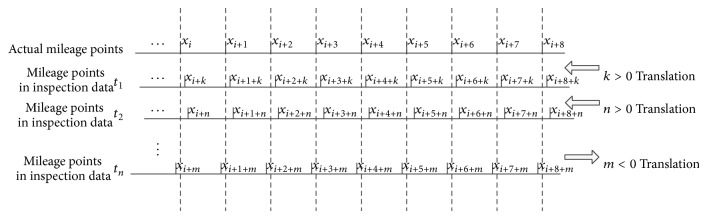
Schematic diagram of mileage absolute calibration.

**Figure 8:**
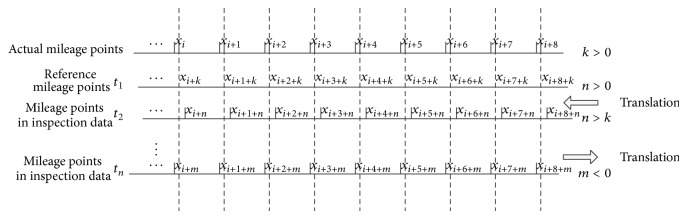
Schematic diagram of mileage relative calibration.

1. Absolute correction refers to the situation when the mileage that each measuring point corresponds to after correction is the accurate mileage. As is shown in Figure 7, the actual mileage data is set for the reference point data, and other data corrects the mileage referring to it. In practice, it needs to know the precise mileage data of the measuring points in precise calibration, but it is difficult to be realized in fact, and it has little significance to research and practical application.
2. The relative correction refers to the situation that all measuring points of each inspection data after correction are pointing at the same mileage. As is shown in Figure 8, each inspection data takes *t*_1_ mileage point data as the reference data, and other data corrects the mileage referring to it. But the mileage point may shift with the actual mileage points.

Both data after the above two types of correction can be used to do the time series data analysis, and there is little difference in practice. The latter is used in this paper.

The goal of mileage correction is to find each measuring point track irregularity status trends over time. Without mileage correction, the correspondent mileage of the all previous inspection data at each correspondent point is not the same with the actual mileage. This is similar to the practice that using the time series data consisted of data at different points to analyze the state changes of a certain point, and this will inevitably lead to inaccurate results.

In this paper, the idea of track space irregularity waveform similarity matching is applied to track irregularity mileage correction of sections. Typically, similarity distance is used to judge the similarity between two sequences. Euclidean distance and non-Euclidean distance are two types of methods to measure the distance.

Because Euclidean distance needs strict correspondence between all points of the sequence in the process of computing, and as a result, the following situation will appear: even a slight shift in the mileage of the inspection data will also make Euclidean distance between the two sections become large. Hence the deficiencies of Euclidean distance needs to be overcome.

In order to solve the problems of drift and noise data in track inspection car mileage data, this paper presents time series correction method based on trend similarity level.

**Figure 9:**
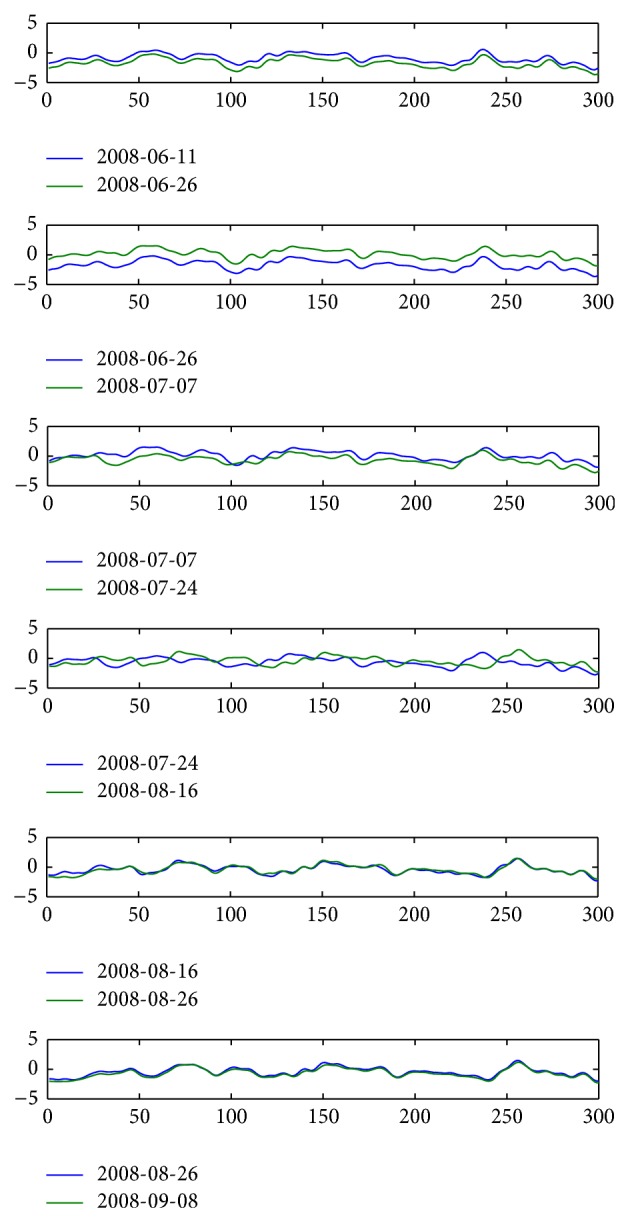
Distribution of gauge irregularity inspection data from February 2, 2008, to June 11, 2008, before mileage correction.

The gauge inspection data in February 20, 2008, to November 13, 2008, Beijing-Kowloon line, section of K500+000–K500+075 km is selected for the study. The distribution of gauge inspection data of two adjacent sections before correction is shown in Figure 9.

**Figure 10:**
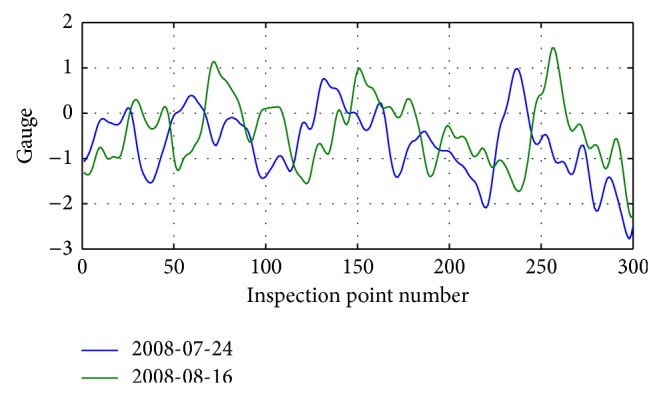
Distribution of gauge irregularity inspection data between July 24, 2008, and August 16, 2008.

The distribution of gauge irregularity inspection data details between two inspections on July 24, 2008, and August 16, 2008, is shown in Figure 10.

As can be seen from Figure 10, the gauge data have a certain offset compared to the corresponding mileage data.

There are three types of changing trends in adjacent track irregularity time series data elements: rising, falling, and flat. While *x*_*j*,*t*_*i*__ > *x*_*j*,*t*_*i*−1__  (1 ≤ *i* − 1 < *i* ≤ *n*), the data changing trend is upward; while *x*_*j*,*t*_*i*__ < *x*_*j*,*t*_*i*−1__  (1 ≤ *i* − 1 < *i* ≤ *n*), the data changing trend is downward; while *x*_*j*,*t*_*i*__ = *x*_*j*,*t*_*i*−1__  (1 ≤ *i* − 1 < *i* ≤ *n*), the data changing trend is flat. As the research is carried out on the same section repeatedly, all inspection data should reflect similar trends of the track irregularity state.

According to the idea of similar trends, data correction on track irregularity time series is done. There are four steps of data correction.

*First Step: Trend Data Transformation*. Gauge irregularity data is selected for the study. Assume the inspection time series data, whose length is *n*, consisted of *n* measurement points in the unit section as follows:

(2) X1=x1,t1,x1,t2,…,x1,ti,…,x1,tn,X2=x2,t1,x2,t2,…,x2,ti,…,x2,tn,⋮Xj=xj,t1,xj,t2,…,xj,ti,…,xj,tn,⋮Xn=xn,t1,xn,t2,…,xn,ti,…,xn,tn.

In this formula, *X*_*j*_ is inspection sequence data formed of the *j*th inspection of the section and *X*_*j*+1_ is inspection sequence data formed of the *j* + 1th inspection of the section. As there is mileage offset in track inspection data, inconsistencies exist in mileages of the measuring points corresponding to the two sequences.

Trend processing methods of data are as follows.

 First, define the trend series *X*_*j*_′, *X*_*j*_′ = (*x*_*j*,*t*_1__′, *x*_*j*,*t*_2__′,…, *x*_*j*,*t*_*i*__′,…, *x*_*j*,*t*_*n*−1__′).
 Then, series *X*_*j*_ is transformed into a series trend *X*_*j*_′.
 When *x*_*j*,*t*_*i*+1__ > *x*_*j*,*t*_*i*__  (1 ≤ *i* ≤ *n* − 1), *x*_*j*,*t*_*i*__′ = 1.
 When *x*_*j*,*t*_*i*+1__ < *x*_*j*,*t*_*i*__  (1 ≤ *i* ≤ *n* − 1), *x*_*j*,*t*_*i*__′ = −1.
 When *x*_*j*,*t*_*i*+1__ = *x*_*j*,*t*_*i*__  (1 ≤ *i* ≤ *n* − 1), *x*_*j*,*t*_*i*__′ = 0.
 The process of sequence *X*_*j*+1_ is the same with *X*_*j*_.

Here, *x*_*j*,*t*_*i*__′ is the element of the trend series *X*_*j*_′, and the length of trend series *X*_*j*_′ is (*n* − 1). Elements of the trend series data *X*_*j*_′ and *X*_*j*+1_′ after conversation are composed of 1, 0, −1, such as

(3) $$\begin{array}{c} X_{j}^{\prime }=\left(1,1,1,-1,1,-1,-1,0,\ldots \right),\\ X_{j+1}^{\prime }=\left(1,1,-1,-1,1,-1,1,0,\ldots \right). \end{array}$$

**Figure 11:**
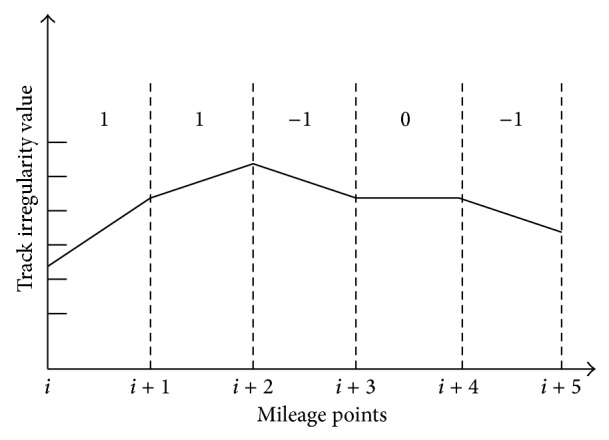
Trend analyses of time series data.

Graphical representation of the process of sequence trend transformation is shown in Figure 11.

*Step Two: Calculate the Similarity of Trend Sequences*. To evaluate the similarity of the trend sequences *X*_*j*_′ and *X*_*j*+1_′, the idea is as follows. When the number of equal corresponding elements between the trend sequences *X*_*j*_′ and *X*_*j*+1_′ is larger, the similarity of trends sequences *X*_*j*_′ and *X*_*j*+1_′ is higher.

*Similarity Level Calculation*. The resulting tendency sequence subtracts from each other to form a new sequence:

(4) $$\begin{array}{c} H=X_{j+1}^{\prime }-X_{j}^{\prime }. \end{array}$$

Assume the number of elements in the sequence *H* is *n* and the number of 0 elements is *h*, and then define the similarity level between the trends sequences *X*_*j*_′ and *X*_*j*+1_′ as

(5) $$\begin{array}{c} l=\frac{h}{n}\times 100\%. \end{array}$$

The larger the number of 0 elements in sequence *H* is, the greater the value *l* is and the higher the similarity of trends sequences *X*_*j*_′ and *X*_*j*+1_′ is. This is the sequence's similarity level before correction.

*Third Step: The Sequence Translation Transformation*. Translation transformation includes left and right translation transformation, in which both left and right are relative to the reference sequence. Take *X*_*j*_′ as reference sequence, left and right translation transformation are carried out. The translation distance is the distance of m measuring and translation distance is set as 0.25 m each time.

(*1) Left Translation Transformation*. Each time when *X*_*j*+1_′ is moved left for a measuring point distance, the operation would amputate the first element of *X*_*j*+1_′ and the last element of *X*_*j*_′. In this case, after elements truncation, the two sequences *X*_*j*_′ and *X*_*j*+1_′ are of equal length. After one step shift operation, elements of the two sequences are corresponding to each other. Next, do the subtraction on the two new sequences, and then calculate the number of zero elements in the sequence formed by subtraction and then calculate the similarity level after the first left translation transformation.

The above process is repeated until *m* measuring points are moved left, and *m* similarity level values are achieved.

(*2) Right Translation Transformation*. The ideological of right translation transformation process is the same with the left. *M* similarity level values can be obtained after *m* times of right translation transformation.

*Step Four: Data Correction*. Trend similarity level values between the original trend sequences before the translation transformation and the similarity level values after *m* left and *m* right translation transformation are selected, and the maximum value of 2*m* + 1 similarity level values is selected as correction criterion, which is as follows in detail.

1. If the trend similarity level before the translation transformation is the maximum, then the sequence needs no correction.
2. When *l*_*lk*_ (*k* steps left) is the maximum value of similarity level in *m* similarity level (*l*_*l*1_, *l*_*l*2_,…, *l*_*lm*_) of the left translation transformation, then *X*_*j*+1_′ and *X*_*j*_′ have the greatest similarity when *X*_*j*+1_′ moves the distance of *k* measuring points to the left. Since *X*_*j*+1_′ and *X*_*j*_′ are obtained by transformation of *X*_*j*+1_ and *X*_*j*_; therefore, the position of *X*_*j*_ and *X*_*j*+1_ has the maximum coherence after *X*_*j*+1_ moves the distance of *k* measuring points to the left, and data mileage between *X*_*j*_ and *X*_*j*+1_ is corrected to be aligned with each other.
3. When *l*_*rp*_ (*p* steps right) is the maximum value of similarity level in *m* similarity level (*l*_*l*1_, *l*_*l*2_,…, *l*_*lm*_) of the left translation transformation, then *X*_*j*+1_′ and *X*_*j*_′ have the greatest similarity when *X*_*j*+1_′ moves the distance of *p* measuring points to the left. Due to the fact that *X*_*j*+1_′ and *X*_*j*_′ are obtained by transformation of *X*_*j*+1_ and *X*_*j*_, therefore, the position of *X*_*j*_ and *X*_*j*+1_ has the maximum coherence after *X*_*j*+1_ moves the distance of *p* measuring points to the right, and data mileage between *X*_*j*_ and *X*_*j*+1_ is corrected to be aligned with each other.

According to experience, the value range of *m* is generally set from 40 to 100.

Two adjacent inspections sequences can be calibrated by translation transformation through finding the position of the maximum value of the similarity level of two adjacent sequences. If the overall mileage data of *n* times inspection data at section is calibrated, a certain time inspection data can be set as a reference data sequence (generally first inspection data is selected), and other sequences do translation transformation according to the position of the maximum value of the similarity level of two adjacent inspection sequences data. The statistics table of similarity level and translation transformation distance is shown in Table 1.

**Figure 12:**
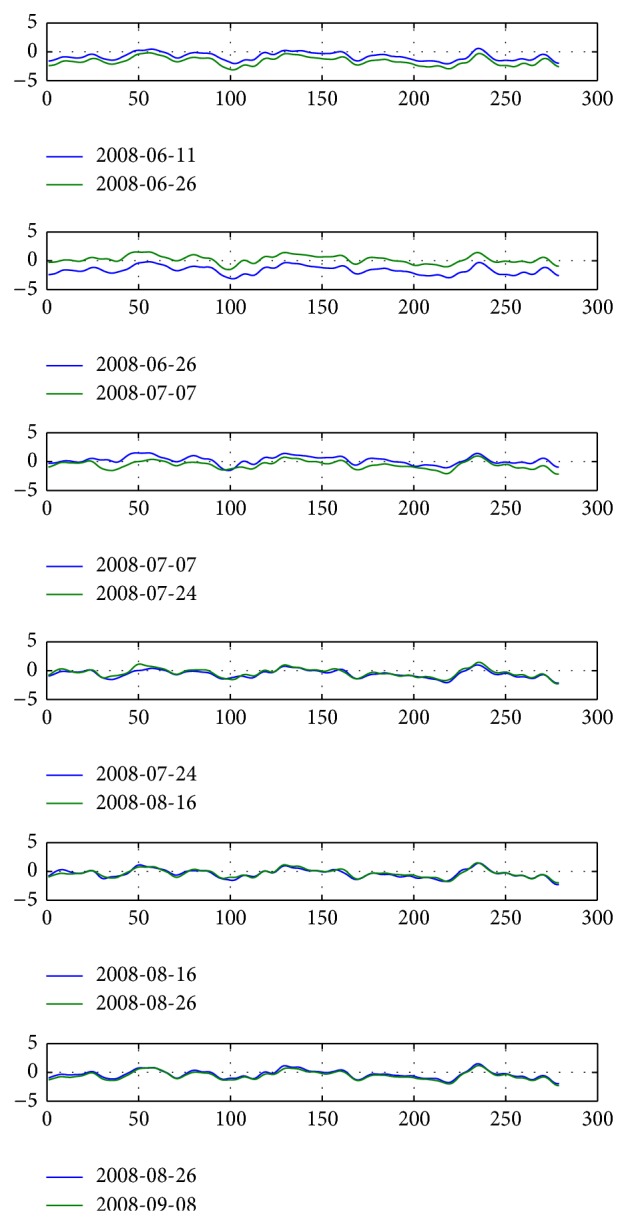
Distribution of gauge irregularity inspection data from February 20, 2008, to June 11, 2008, after mileage correction.

After calibration, the distribution of two adjacent gauge inspection data of sections is shown in Figure 12.

**Figure 13:**
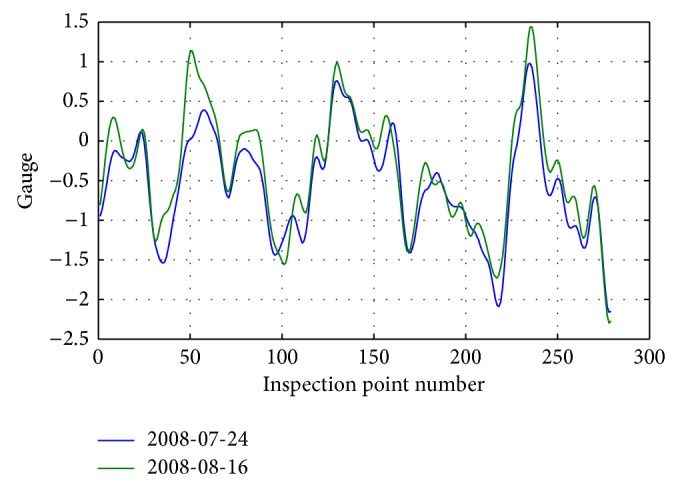
Distribution of details of correction of gauge irregularity inspection data between July 24, 2008, and August 16, 2008.

Distribution of gauge irregularity data of July 24, 2008 and August 16, 2008 is shown in Figure 13.

It should be noted that the mileage offset correction in this study here is a relative correction, because the first inspection sequence is set as a reference sequence in the correction process, and the mileage data is assumed to be with no offset. But the reality is that there is also mileage offset of the reference sequence compared to real mileage data. Therefore, this calibration process belongs to relative correction, but each inspection data is aligned with each other after calibration, which will provide a data base for the research of each measurement point or smaller section within the time track status changes in the following studies.

## 6. Track Irregularity Time Series Data Wavelet Decomposition-Reconstruction

The wavelet transform [28–31] is a new rapidly evolving field of applied mathematics and engineering disciplines; it is a new branch of mathematics, which is the perfect crystal of functional analysis, Fourier analysis, sample transfer analysis, and numerical analysis. Data process or data series is converted into stages data series to find similar spectrum characteristics based on some special functions in the wavelet transform, so as to achieve a data processing. Wavelet transform is local transformation of space (time) and frequency, and it can effectively extract information from the signal and do multiscale detailed analysis to function or signal through stretching and panning arithmetic.

“Wavelet” means the waveform with a small area, the limited length and 0 mean, in which “small” refers to the wavelet with decay, “wave” refers to its volatility, and its amplitude shocks in alternating positive forms and negative forms. Compared with the Fourier transform, wavelet transform is the localized analysis of the time (space) frequency. It does multistage subdivision gradually through stretching shift operation on the signal (function) and ultimately achieves time segments at high frequency and frequency segments at low frequency and can automatically adapt to the requirements of time-frequency signal analysis, and then can focus on any detail of the signal and thus can solve the difficult problem of Fourier transform. It has become a major breakthrough in the scientific method since Fourier transform, so wavelet transform is even called “mathematical microscope”.

The decomposition of the function into the representation of a series of simple basis functions has an important significance both in theory and in practice. In this paper, Daubechies wavelet [32, 33] is used to do decomposition in track irregularity time series data, which is the general term for a series of binary proposed by the French scholar Daubechies, and multiscale wavelet decomposition of the signal can be done by it.

Assume a known signal

(6) $$\begin{array}{c} f\left(x\right)=\sum a_{j,k}\phi_{j,k}\left(x\right),\;f\left(x\right)\in V_{j}. \end{array}$$

The coefficients {*a*_*j*,*k*_, *k* ∈ *Z*} are known in the formula.

Now *f*(*x*) is decomposed into two components of space *V*_*j*−1_ and space *W*_*j*−1_:

(7) $$\begin{array}{c} f\left(x\right)=\sum a_{j-1,k}\phi_{j-1,k}\left(x\right)+\sum d_{j-1,k}\psi \left(x\right). \end{array}$$

In a given situation of sequence {*a*_*j*,*k*_}, respectively, the (*J* − 1)th approximate level sequence {*a*_*j*−1,*k*_} and (*j* − 1)th details level sequence {*d*_*j*−1,*k*_} can be calculated. According to two scale relations, it can be known that

(8) ϕj−1,k=2j−1/2ϕ2j−1x−k=2j−1/22∑shsϕ22j−1x−k−s=∑shs2j/2ϕ2jx−2k+s=∑shsϕj,2k+sx.

Similarly, it can be calculated that

(9) $$\begin{array}{c} \psi_{j-1,k}\left(x\right)=\underset{s}{\sum }g_{s}\phi_{j,2k+s}\left(x\right). \end{array}$$

It can be inferred according to the above relation that

(10) aj−1,k=fx,ϕj−1,k(x)=fx,∑shsϕj,2k+s(x)=∑sh−sfx,ϕj,2k+sx=∑sh−saj,2k+s=∑aj,nh−n−2k=aj×h′2k.

In the formula, $h_{k}^{\prime }={\overset{-}{h}}_{-k}$.

Similarly, it can be calculated that

(11) $$\begin{array}{c} d_{j-1,k}=a_{j}\times g^{\prime }\left(2k\right),\;\;g_{k}^{\prime }={\overset{-}{g}}_{-k}. \end{array}$$

The second level decomposition {*a*_*j*−2,*k*_} and {*d*_*j*−2,*k*_} can be obtained after doing decomposition on the approximate sequence {*a*_*j*−1,*k*_} resulted from the first stage of decomposition results again, and the third level decomposition {*a*_*j*−3,*k*_} and {*d*_*j*−3,*k*_} can be obtained after doing decomposition on the approximate sequence {*a*_*j*−2,*k*_} resulted from the second stage of decomposition results, and so on, until the multiscale wavelet is decomposed into a specified stage.

**Figure 14:**
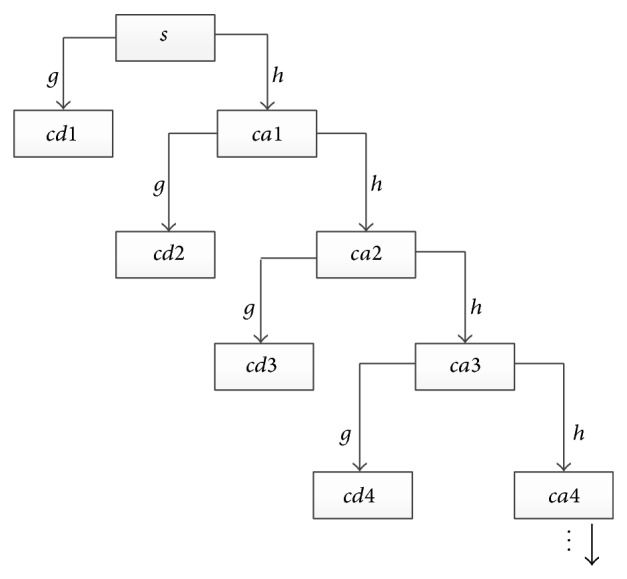
Process of wavelet decomposition.

**Figure 15:**
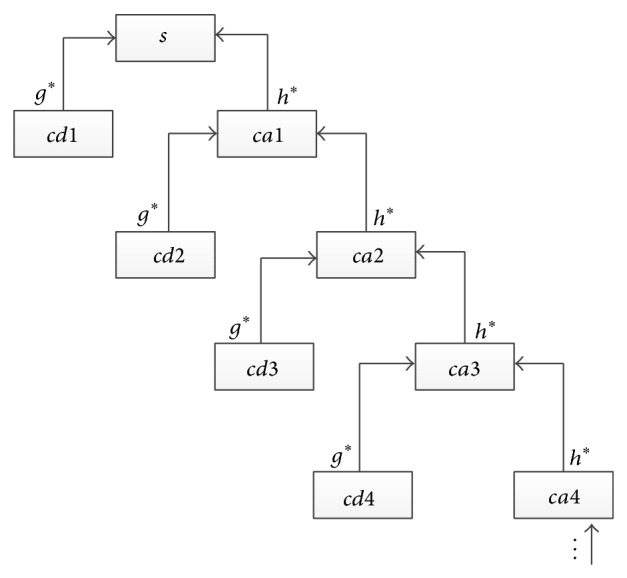
Process of wavelet reconstruction.

The decomposition process is called Mallat Pyramidal algorithm as shown in Figure 15. Mallat algorithm is inspired by the famous Pyramidal algorithm [34] for image decomposition and combined with multiresolution analysis, proposing signal tower multiresolution decomposition and synthesis algorithms. It is named after the data structure which is a tower structure in decomposition process. Decomposition and reconstruction process is shown in Figures 14 and 15.

*S* is the original signal in figure, *cd*1 and *ca*1 are detail sequence and approximate sequence after level 1 decomposition, and *cd*2 and *ca*2 are detail sequence and approximate sequence after level 2 decomposition, and so on.

Standard deviation reflects changes of the deviation from mean of track irregularity. When distribution of irregularity around the mean value is more discrete, the representation of average is poorer, and track irregularity will be in poorer state. Conversely, the smaller the standard deviation is, the smaller the variation between the track irregularity values is, the denser the irregularity distribution around the mean value is, and the better the representation of the mean and track state is.

**Figure 16:**
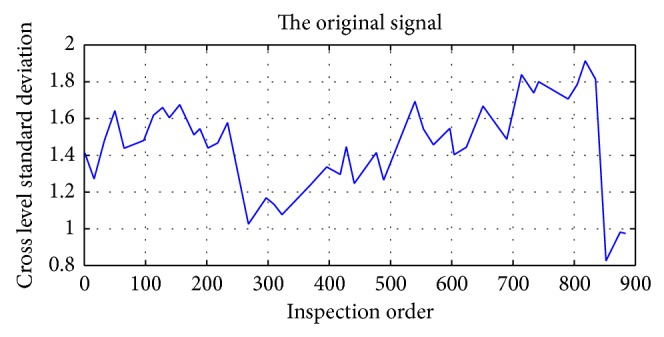
Original waveform signal of track irregularity.

Changes of track irregularity standard deviation series data of the Beijing-Kowloon line K449+000–K450+000 sections within 44 times inspection are selected as the research object, and the original signal is shown in Figure 16.

Daubechies wavelet is chosen in signal decomposition of track irregularity standard deviation time series data, with the decomposition depth 3. Mallat tower algorithm is used for decomposition and reconstruction of track irregularity standard deviation time series. After wavelet decomposition, 1, 2, and 3 layers are the waveform signal (high frequency) of details, respectively, represented by *D*1, *D*2, and *D*3; and approximate sequence waveform signal (LF) of layer 3 is represented by *A*3.

**Figure 17:**
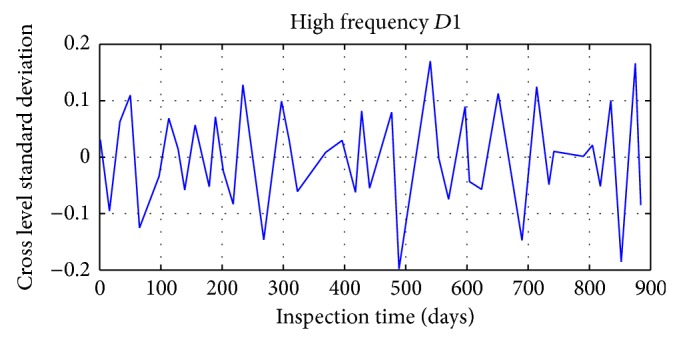
The first layer detail waveform signal of track irregularity (HF).

**Figure 18:**
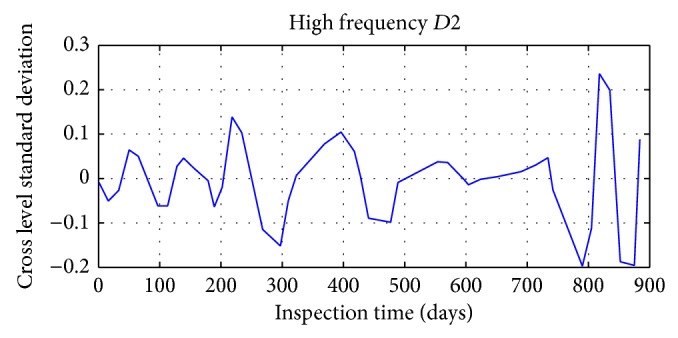
The second layer detail waveform signal of track irregularity (HF).

**Figure 19:**
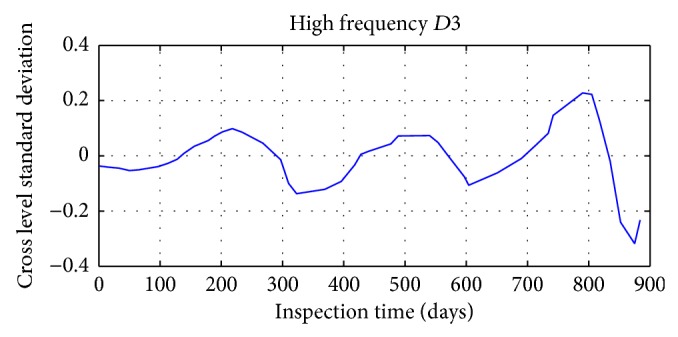
The third layer detail waveform signal of track irregularity (HF).

**Figure 20:**
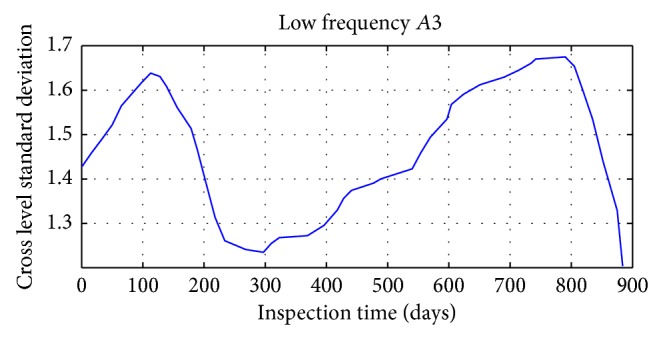
The third layer approximation waveform signal of track irregularity (LF).

The results of specific decomposition are shown in Figures 17, 18, 19, and 20.

All layers waveform signals after decomposition are reconstructed with a weight of 1, and the reconstruction formula is as follows:

(12) $$\begin{array}{c} s=ca3+cd3+cd2+cd1. \end{array}$$

**Figure 21:**
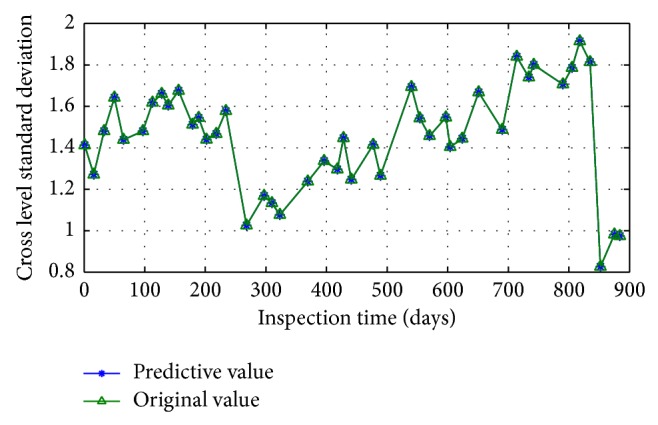
Comparison between original data sequence and reconstructed data sequence.

Reconstruction results are shown in Figure 21.

**Figure 22:**
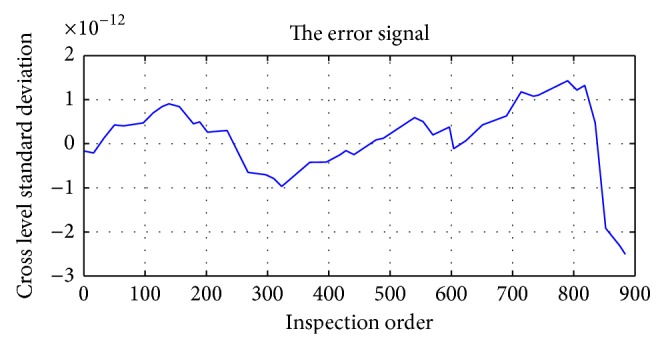
Data error.

Error analysis is shown in Figure 22.

Error analysis showed that when reconstruction is used by weight of 1, the order of error will be 10^−12^, which is basically negligible.

It is very important to study the wavelet decomposition-reconstruction of track irregularity data. After wavelet decomposition, track irregularity time series data can be transformed into multifeature smooth sequence from nonstationary characteristics, which is an effective data preprocessing method in time series modeling with the premise for a smooth sequence. By wavelet decomposition, further clarification can be done to the characteristics of data changes and thus can provide a basis for classification, clustering, and pattern recognition. Meanwhile, by modeling and analysis on data at each layer, respectively, optimal fit and predictive models can be obtained, and then we can carry out weighted calculation to models of all layers and then get fit and predicted values of the original track irregularity time series data.

## 7. Change Mode of Unit Section

It is less meaningful to study track state changes of a fixed inspection point; based on the tools and interval of data collection, it is of great significance to study the state changes of the overall length of certain sections.

Track Irregularity inspection data appears near zero mean, positive and negative phases alternatively. There is a strong stochastic changing characteristic of each measuring point in track irregularity state inspection process. Character of track irregularity state in a single measuring point position showed that track irregularity track geometry data fluctuate on the standard values, but this variable is a random process, with the direction and the size changing from time to time, and the real trend of track state changes cannot be reflected. Therefore, irregularity size change in a single direction and magnitude of a single track geometry measurement points should not be seen as the basis in the study. The distribution deviating from the normal value and the rate of development of the unit section should be used to measure changes of track irregularity values.

In summary, to study the features of a certain length of section track irregularity state changes, the standard deviation of track irregularity inspection data can be used as the object in study.

**Figure 23:**
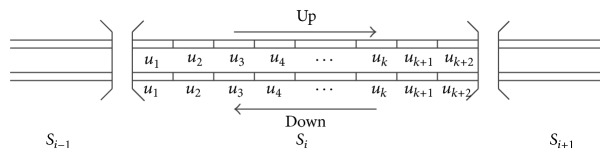
Selection of study data.

Take the 44 times' inspection data of the cross level and longitudinal track irregularity, Beijing-Kowloon line K449+000–K450+000 section, in 884 days, between February 20, 2008, and July 23, 2010, as the study data. The section is divided into 40 unit sections, and each data contain 100 measure points, and the track state changes of each unit section are studied. The way of data selection is shown in Figure 23.

*S*_*i*−1_, *S*_*i*_, and *S*_*i*+1_ are division of unit section in study in figure, 1 km is selected as the length of unit section in this paper, and *u*_1_, *u*_2_,… are unit sections divided in the study.

**Figure 24:**
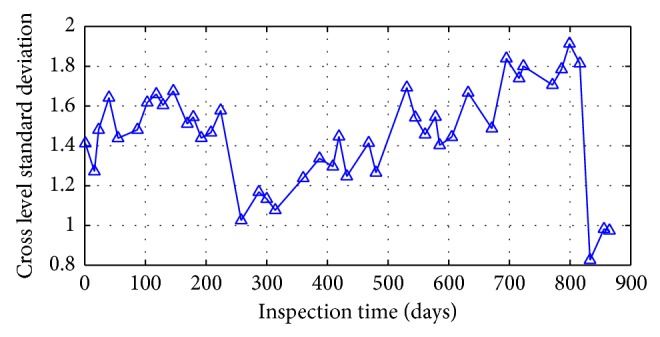
Cross level status trends at unit section of K449+200–K449+225.

**Figure 25:**
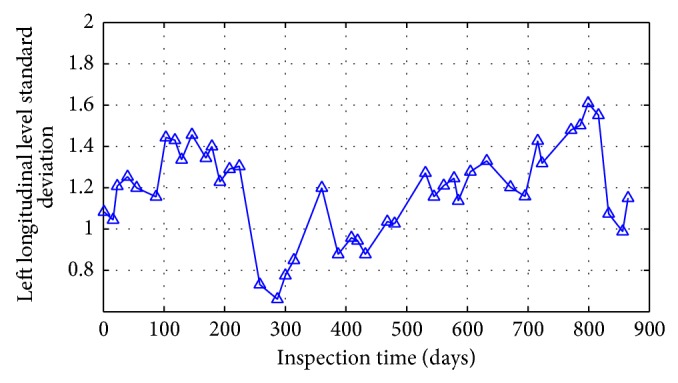
Left longitudinal level status trends at unit section of K449+675–K449+700.

According to the data selection methods above, track irregularity standard deviation data of some unit sections is shown in Figures 24 and 25.

There are two types of changing modes presented in the standard deviation curve in the changing process over time from the analysis of Figures 24 and 25.

(*1) Jump*. In the adjacent inspection time *t*_*i*−*n*_,…, *t*_*i*_, *t*_*i*+1_,…, *t*_*i*+*m*_, *sd*_*i*−*n*_,…, *sd*_*i*_, *sd*_*i*+1_,…, *sd*_*i*+*m*_ are track irregularity standard deviations corresponding to them, when

(13) sdi+1−sdi≫sdi−sdi−1,sdi+1−sdi≫sdi−1−sdi−2,⋮sdi+1−sdi≫sdi+2−sdi+1,sdi+1−sdi≫sdi+3−sdi+2,⋮

This phenomenon of standard deviation curves is considered to be showing jump change in the adjacent time *t*_*i*_, *t*_*i*+1_. The reason for the jump changes in the time *t*_*i*_ is that track state degradation reaches a critical value of maintenance, and the maintenance operation is imminent; the track condition is significantly becoming better in time *t*_*i*+1_, showing that the track has undergone maintenance operations. This jump change is the demarcation point of track status cycle change.

(*2) Gradual Variation*. In the adjacent inspection time *t*_*i*−*n*_,…, *t*_*i*_, *t*_*i*+1_,…, *t*_*i*+*m*_, *sd*_*i*−*n*_,…, *sd*_*i*_, *sd*_*i*+1_,…, *sd*_*i*+*m*_ are track irregularity standard deviations corresponding to them, when

(14) sdi+1−sdi≈sdi−sdi−1,sdi+1−sdi≈sdi−1−sdi−2,⋮sdi+1−sdi≈sdi+2−sdi+1,sdi+1−sdi≈sdi+3−sdi+2,⋮

This phenomenon of standard deviation curves is considered to be showing gradual changes in the adjacent time *t*_*i*_, *t*_*i*+1_. The reason for the gradual change is that the track changes in a steady state at the moment of *t*_*i*_ and the adjacent time, indicating that track changes are in a maintenance cycle currently.

**Figure 26:**
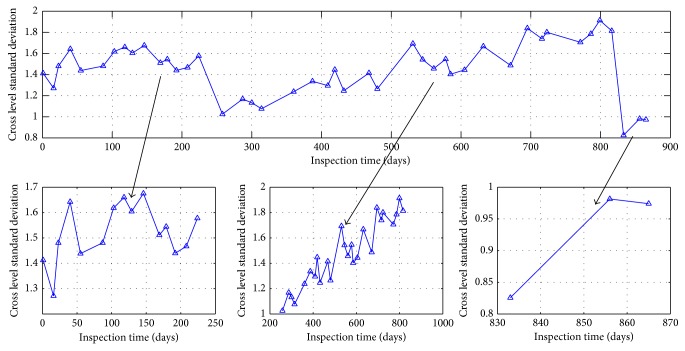
Cycle decomposition of cross level trend at unit section of K449+800–K449+825.

It can be considered that the changes of cross level standard deviation and left longitudinal level standard deviation show a periodic growth pattern through the curve geometric features in Figures 24 and 25. Take the changes of cross level standard deviation state at K449+800–K449+825 unit section as the example; the changing trend of track irregularity state characters in 884 days is divided by the two jump models at 268th days and 835th days into three cycles. Among this, it is a complete changing cycle between the 268th days and the 835th days. The cycle is shown in Figure 26 periodically.

Cyclical characteristics result from the operation of railway maintenance. Due to regular track irregularity maintenance and repair work, as well as the timely remediation repair in cases of overrun, the trend curve of track irregularity often shows a number of jumping phenomena. Also, since this maintenance and repair work are conducted at specific locations, for example, they are operated within tens of meters, a few hundred meters, and even several kilometers and the difference of irregularities and targets in remediation operations, for example, one or a number of remediation to cross level and longitudinal level, thus in the specific object section study, the cyclical nature of the state reflected by each single irregularity inspection will be different.

## 8. Conclusions

The characteristics of track irregularity data are systematically analyzed in this paper. Targeted on the problems of data quality, data offset correction algorithm is proposed based on trends similarity, as well as the outlier identification and noise cancellation algorithms based on the abnormal degree, so as to do treatment on data. Next, the paper proposes track irregularity time series decomposition and reconstruction by using the wavelet decomposition and reconstruction approach. Finally, since the data of track geometry irregularity reflect dynamic changing characteristics of the track state, as a result, through the research on pattern features of track irregularity standard deviation data series of the section, the changing trends of data is discovered and described. The model proposed in this paper is a general model and model can be used in most cases. The results can provide a theoretical basis for subsequent track condition predictions.

## Figures and Tables

**Table 1:** Statistics table of similarity level and mileage correction distance.

Number	Adjacent inspection time	The maximum similarity level	Translational direction	Translation distance	Overall translation distance
1	2008-02-20 and 2008-03-06	235/299	Left	2	Left 2
2	2008-03-06 and 2008-03-23	270/299	Right	1	Left 1
3	2008-03-23 and 2008-04-09	267/299	Left	1	Left 2
4	2008-04-09 and 2008-04-24	264/299	Right	1	Left 1
5	2008-04-24 and 2008-05-26	243/299	Inalienable	0	Left 1
6	2008-05-26 and 2008-06-11	247/299	Left	1	Left 2
7	2008-06-11 and 2008-06-26	256/299	Left	1	Left 3
8	2008-06-26 and 2008-07-07	239/299	Left	1	Left 4
9	2008-07-07 and 2008-07-24	231/299	Right	2	Left 2
10	2008-07-24 and 2008-08-16	220/299	Left	19	Left 21
11	2008-08-16 and 2008-08-26	232/299	Inalienable	0	Left 21
12	2008-08-26 and 2008-09-08	254/299	Inalienable	0	Left 21
13	2008-09-08 and 2008-09-24	254/299	Right	4	Left 17
14	2008-09-24 and 2008-10-10	260/299	Left	1	Left 18
15	2008-10-10 and 2008-11-13	234/299	Inalienable	0	Left 18
16	2008-11-13 and 2008-12-12	246/299	Inalienable	0	Left 18
17	2008-12-12 and 2008-12-25	244/299	Inalienable	0	Left 18
